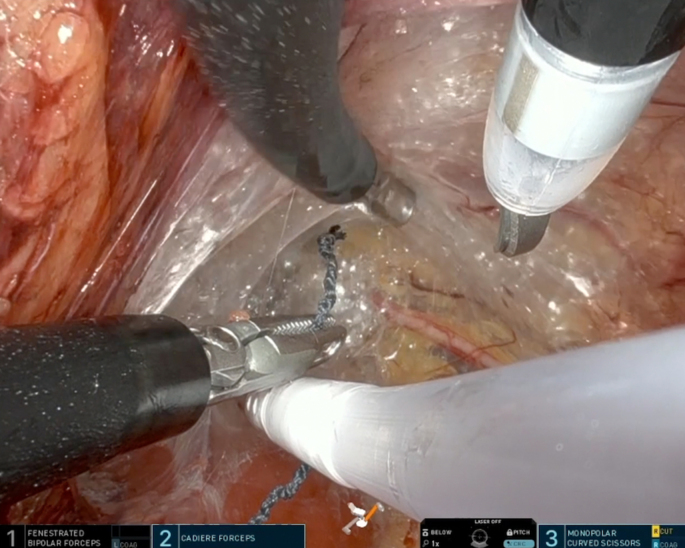# Single-port robot assisted partial nephrectomy via the supine anterior retroperitoneal approach (SARA)

**DOI:** 10.1016/j.eucr.2025.103002

**Published:** 2025-03-03

**Authors:** Sij Hemal, Sina Sobhani

**Affiliations:** USC Norris Comprehensive Cancer Center, Institute of Urology, Keck School of Medicine of University of Southern California, Los Angeles, CA, USA

**Keywords:** Single port, Urology, Partial nephrectomy, Supine anterior retroperitoneal access

## Abstract

This video explores the technique of robot-assisted partial nephrectomy using the Da-Vinci Single-Port robot via SARA in a 56-year-old male with history of diverticulitis found to have a 2.5 cm renal mass upon workup for abdominal pain. Retroperitoneal access was obtained at the McBurney point for port placement. Surgical steps: 1) retroperitoneal access at McBurney's point 2) renal hilum dissection 3) renal tumor identification 4) intraoperative ultrasound 5) hilar clamping 6) renal tumor excision using enucleoresection technique 7) Deep renorrhaphy 8) Early unclamping and cortical renorrhaphy. Surgery was successful without any complications with a warm ischemia time of 14 minutes.

## 1 Introduction

The DaVinci Single Port (SP) robotic platform simplifies access to a confined retroperitoneal space potentially facilitating retroperitoneoscopic surgery. In report, we present robot-assisted partial nephrectomy for a cT1a right renal mass using the Supine Anterior Retroperitoneal Approach (SARA).^1^, ^2^, ^3^, ^4^, ^5^, ^6^, ^7^, ^8^, ^9^

## 2 Case presentation and technique

**Fig. 1:**
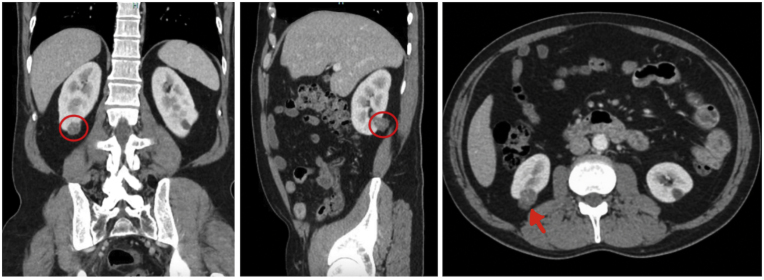
CT views of the right kidney tumor (encircled) with a slight bulge toward the sinus fat.

56-year-old male with a body mass index (BMI) of 27 and a history of diverticulitis was referred for management of an incidental 2.5 cm right renal mass discovered on workup of abdominal pain. The right kidney mass can be viewed in his CT scan in Fig. 1. His past medical and family history was non-contributory and metastatic workup was negative.

**Fig. 2:**
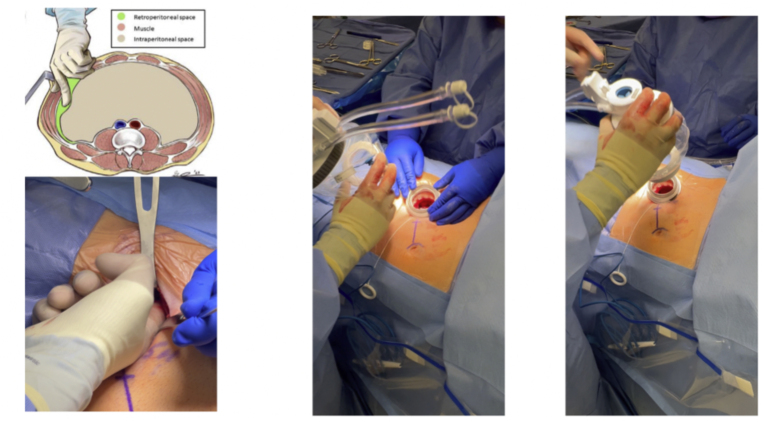
Insertion of the single-port access kit following retroperitoneal access in the Supine Anterior Retroperitoneal Approach.

The patient was placed in a supine position, with 10–20-degree elevation of the ipsilateral flank. Retroperitoneal access was obtained, starting with an incision at the McBurney's point. Using a muscle-splitting technique, dissection proceeded through Camper's and Scarpa's fascia until the external oblique fascia was visualized. An incision was made on the external oblique fascia, and blunt dissection continued through the internal and external oblique and transversalis muscles, allowing entry into the retroperitoneal space. Finger dissection was used to expand the retroperitoneal space lateral to the peritoneum, extending until the ipsilateral anterior superior iliac spine was palpable. Gentle finger dissection was performed medially to push the peritoneum away from the transversus abdominis muscle, creating sufficient space for the insertion of the single-port access kit (Fig. 2).

**Fig. 3:**
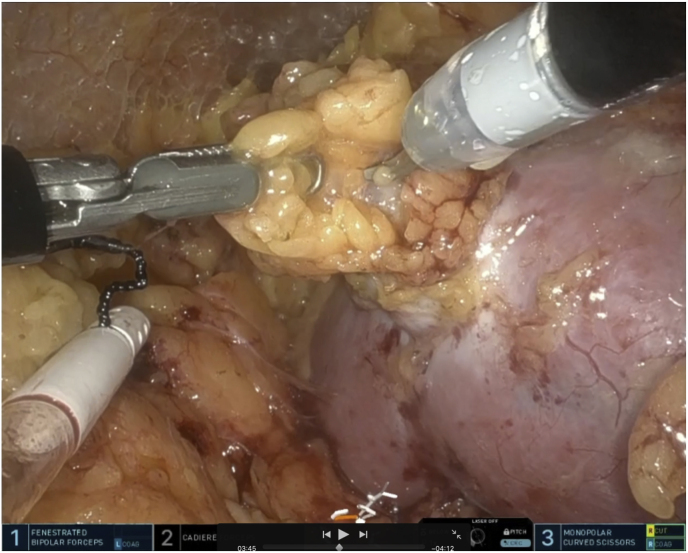
Renal tumor excision in posterior aspect of the interpolar region of the right kidney after defatting the kidney.

Primary surgeon (SH) performed the procedure using the SARA. Surgical steps demonstrated: 1) access to retroperitoneal space obtained at McBurney's point 2) dissection of renal hilum 3) identification of renal tumor 4) intraoperative ultrasound 5) hilar clamping 6) renal tumor excision using enucleoresection technique 7) Deep renorrhaphy 8) Early unclamping and cortical renorrhaphy (Fig. 3, Figure 4, Video 1).^1^^,^^10^^,^^11^

Supplementary data related to this article can be found online at doi:10.1016/j.eucr.2025.103002

## 3 Results

Surgery was successful without any complications with a warm ischemia time of 14 minutes and estimated blood loss of 50 mL. Total console time was 100 minutes. Early unclamping technique was utilized to ensure hemostasis. No drain was placed. The patient was discharged on post-operative day one. Final pathology revealed a 2.6 cm clear cell renal cell carcinoma (pT1a) with negative surgical margins.

## 4 Discussion and conclusions

SP robot-assisted retroperitoneal partial nephrectomy confers similar benefits achieved by conventional laparoscopic and multi-port robotic retroperitoneoscopic renal surgery while achieving equivalent oncologic and functional outcomes. Furthermore, the efficient articulation of robotic instruments through a single incision in a confined space may mitigate ergonomic challenges posed by a bulky multi-port robotic or laparoscopic platform while replicating the benefits of retroperitoneoscopic renal surgery.

## CRediT authorship contribution statement

**Sij Hemal:** Writing – review & editing, Writing – original draft, Visualization, Validation, Supervision, Methodology, Data curation, Conceptualization. **Sina Sobhani:** Writing – review & editing, Writing – original draft, Visualization, Supervision, Software, Project administration, Methodology, Conceptualization.

## Patient consent statement

A written informed consent for the publication of this case was obtained from the patient.

## Funding

None.

## Declaration of competing interest

The authors declare that they have no known competing financial interests or personal relationships that could have appeared to influence the work reported in this paper.

## Figures and Tables

**figs1:**